# Impact of Varicella Immunization and Public Health and Social Measures on Varicella Incidence: Insights from Surveillance Data in Shanghai, 2013–2022

**DOI:** 10.3390/vaccines11111674

**Published:** 2023-11-01

**Authors:** Liming Shi, Jia Lu, Xiaodong Sun, Zhi Li, Liping Zhang, Yihan Lu, Ye Yao

**Affiliations:** 1 School of Public Health, Fudan University, 131 Dong’an Road, Shanghai 200032, China; slmioe@163.com (L.S.); luyihan@fudan.edu.cn (Y.L.); 2 Key Laboratory of Public Health Safety, Fudan University, Ministry of Education, 131 Dong’an Road, Shanghai 200032, China; 3 Minhang District Center for Disease Control and Prevention, 965 Zhongyi Road, Shanghai 201101, China; cpulj@126.com (J.L.); zhanglp828@163.com (L.Z.); 4 Shanghai Municipal Center for Disease Control and Prevention, 1380 West Zhongshan Road, Shanghai 200336, China; sunxiaodong@scdc.sh.cn (X.S.); lizhi@scdc.sh.cn (Z.L.)

**Keywords:** varicella, incidence, vaccination program, PHSMs

## Abstract

To evaluate the impact of a two-dose VarV program on varicella incidence among the whole population, considering the influence of public health and social measures (PHSMs), we extracted surveillance data on varicella cases during 2013–2022 in Minhang, Shanghai. Then, we estimated the incidence trend of varicella through interrupted time-series analyses and quantified the impact of the immunization program and PHSMs using Serfling regression. We also explored the associations between PHSMs and varicella cases. The implementation of the two-dose VarV strategy was followed by a significant decrease in varicella incidence (−1.84% per month). After one year of the program, varicella incidence was estimated at a 45.25% reduction, which was higher in children (59.12% and 54.09%) than in adults (19.49%). The decrease attributed to PHSMs was 31.26% during 2020–2022, and school closing was identified as the most relevant PHSM (b = −8.03 cases, r = −0.67 with a 1-week lag). These findings indicate that the two-dose immunization program has more effectively reduced the varicella incidence compared with the one-dose vaccine, and interventions like school closings are also encouraged to serve as supplementary measures to prevent varicella epidemics.

## 1. Introduction

Varicella is highly infectious, with the secondary attack rate in susceptible contacts ranging from 61% to 100% [1,2]. Although varicella usually leads to mild symptoms, serious complications, including bacterial sepsis, pneumonia, encephalitis, and even death, may occur [3,4,5]. It has been reported that about 268 out of 100,000 varicella patients are hospitalized, and 9.22 per 100,000 patients die [6,7]. Recently, the World Health Organization (WHO) estimated that there were at least 140.0 million cases of varicella globally each year, causing 4.2 million serious complications and 4200 related deaths [8]. In China, varicella is the third most frequently reported vaccine-preventable infectious disease, with approximately one million cases [9]. As varicella is not officially included in notifiable infectious diseases, the real burden of varicella might be underreported. In Shanghai, however, varicella has been monitored since 2006 [10]. Local health providers are required to report varicella cases within 24 h via the surveillance system. After more than eight years of regulation, it is unlikely that the surveillance data would underestimate the actual incidence.

Active immunization is considered one of the most effective ways to prevent varicella. The varicella vaccine (VarV) was first approved for use in the United States in 1995, and varicella cases declined by 71–84% within 5 years in surveillance areas [11]. Although a one-dose VarV is highly effective, varicella outbreaks and breakthrough infections continue to occur in vaccinated populations [12,13]. A two-dose vaccine schedule can further reduce the varicella burden and prevent outbreaks, with an effectiveness of 92% against all varicella cases and 100% against severe complications [14,15]. The WHO recommends universal vaccination in places where varicella remains a public health problem [8]. However, the VarV has not been included in national immunization programs in many countries at present. Since 1999, Shanghai has introduced voluntary self-paid VarV. In 2017, Shanghai adjusted varicella vaccination into a two-dose procedure, and the two-dose VarV has been included in the local immunization program since August 2018 [16]. In this program, children aged 12–18 months will go to a local community health service center for the first free dose of VarV, and the second free dose will be offered to them at 4 years of age. There is also a vaccination scheme for children who have not been vaccinated in a timely manner.

Apart from varicella vaccinations, public health and social measures (PHSMs), such as school closures and home isolation for infected individuals, also help to control the spread of varicella [17]. A previous study estimated that student contact was 22% to 31% lower during the summer holiday than during school time, which led to a lower rate of varicella transmission [18]. Another study showed that the cumulative number of cases dropped to 13 from 35 when home isolation started with the first discovery of patients [17]. During the COVID-19 pandemic, a series of PHSMs aimed at suppressing COVID-19 transmission were implemented [19,20]. These measures are well documented in the Oxford COVID-19 Government Response Tracker database (OxCGRT), making it possible to study the effect of PHSMs on diseases of interest [21].

In this study, the main objective was to evaluate the impact of a two-dose VarV immunization program on varicella incidence using surveillance data from 2013 to 2022 in Minhang, Shanghai. The impacts were quantified through interrupted time-series analysis and Serfling regression. In addition, as PHSMs were implemented to control the COVID-19 pandemic during 2020–2022, this study also analyzed the impact of related PHSMs on varicella incidence. These results could provide evidence for introducing a two-dose VarV strategy and taking effective measures to control varicella epidemics.

## 2. Materials and Methods

### 2.1. Study Setting

Minhang District is situated in the middle of Shanghai Municipality, China, spanning across the inner, central, and outer ring roads of the city geographically. Minhang has approximately 2.65 million residents, which account for 10% of the Shanghai population. The demographic features like age structures in Minhang are consistent with those in Shanghai according to the national census. In addition, as the disease surveillance system, immunization program, and PHSMs in the pandemic in Minhang District are implemented in accordance with the relevant regulations of Shanghai Municipality, the database included in this study can be a good representation of Shanghai. Since 2006, varicella has been managed as a mandatory reporting disease in Shanghai. Local health providers and doctors are required to fill in the infectious disease report form once they diagnose patients with varicella and report to the district Center for Disease Control and Prevention (CDC) within 24 h via the surveillance system. This report form follows a national standard format and records the epidemiologic information for cases: age, sex, occupation, current home address, symptoms onset date, and diagnosis date. In 2017, varicella vaccination was adjusted into a two-dose procedure in Shanghai and has been included in the local immunization program since August 2018. The vaccination records of children are registered on the Minhang CDC Vaccine Management and Vaccination Service System, including demographic and vaccination information.

### 2.2. Data Sources

The surveillance data of reported varicella cases in Minhang, Shanghai, during 2013–2022 were obtained from the Minhang CDC. In consideration of the influence of vaccination, the varicella vaccination data for birth cohorts from 2013 to 2018 were also extracted from the Minhang CDC Vaccine Management and Vaccination Service System.

The two-dose VarV program was implemented in Shanghai in 2017 and has been included in local immunization program since August 2018. In this program, the first dose of the VarV is freely offered to children at 12–18 months, and the second dose is for children 4 years of age.

The Oxford COVID-19 Government Response Tracker (OxCGRT) database has recorded national- and provincial-level government response data to the COVID-19 pandemic since 1 January 2020 [21]. The OxCGRT database contains 19 indicators of government response policies, including policies related to containment and closures, health systems, economies, and vaccinations. The PHSMs data in Shanghai from January 2020 to December 2022 were extracted from the database. The indicators included in this study were school closing, workplace closing, cancellation of public events, restrictions on gatherings, public transportation closures, stay-at-home requirements, restrictions on internal movement, restrictions on international travel, and facial coverings. These indicators were tested for multicollinearity using variance inflation factors and were found not to be collinear with each other.

According to the implementation of the VarV strategy and PHSMs, the following three periods were defined: the one-dose VarV period, from January 2013 to October 2017 (before the two-dose VarV strategy); the early two-dose VarV period, from January 2018 to December 2019 (implementation of the two-dose VarV strategy); and the pandemic period, from February 2020 to December 2022 (PHSMs implemented for COVID-19).

To calculate varicella incidence per 100,000 inhabitants, population data were obtained from the Shanghai Statistical Yearbook as a denominator [22], and the number of reported cases in surveillance data was a numerator.

### 2.3. Statistical Analysis

Interrupted time-series analysis was conducted to estimate changes in the incidence trends of varicella before and after the implementation of the two-dose VarV strategy and PHSMs [23,24]. Seasonality was taken into account using an additive model, and the remaining autocorrelation was tackled using an autoregressive moving average term. November to December 2017 was considered a transition period, during which a two-dose VarV program was introduced. January 2020 was also considered a transition period because PHSMs implementation started in this month. The validity of the segmented regression was assessed by visual inspection of the correlograms and residual analysis. Sensitivity analyses were performed without excluding these transition periods.

Serfling regression was used to quantify the impact of the two-dose VarV strategy and PHSMs, which has been frequently employed in research on communicable diseases [25,26,27]. Based on the monthly incidence of varicella in Minhang during 2013–2017, predictions using Serfling regression were made on the incidence from 2018 through 2022, which was further compared with the actual data to determine the effects of the two-dose VarV strategy after 2017 and PHSM implementation during the COVID-19 pandemic on varicella incidence.

The relationship between each PHSM indicator and the number of weekly varicella cases was examined using cross-correlation Spearman rank correlation analyses, where case figures were time-shifted against PHSM data by 10 weeks. To address the potential effect of covariates, partial correlation analyses and multiple regression were performed.

A two-tailed *p* value less than 0.05 was considered statistically significant. False discovery rate (FDR) correction was performed to account for multiple testings. All statistical analyses were performed in 2023 using R software (version 4.1.3).

## 3. Results

### 3.1. Characteristics of Varicella Cases

From 2013 through 2022, 19,593 varicella cases were reported in Minhang District (median (IQR) age, 11 [5–24] years; 10,656 (54.39%) males and 8937 females (45.61%)) (Table 1). Children aged 5–19 years accounted for 48.51% of the cases, and adults aged ≥20 years accounted for 33.14%. More than half (56.93%) of the cases were reported in students in kindergartens, elementary schools, etc. Varicella cases were reported throughout the period and had a seasonal pattern with two peaks (March to May and October to January) and two troughs (February and August to September) in Minhang. During the early two-dose VarV period, the median age of cases increased from 9 years to 12 years. The number of cases decreased in all age groups, whereas the proportion of cases in adult groups aged 20 years and older increased to varying degrees. In all three periods, there were more cases in men than in women, with sex ratios ranging from 1.15 to 1.21.

### 3.2. Coverage Rates of Varicella Vaccination among Children

The coverage rate of the first dose of VarV ranged from 92.67% in the 2018 birth cohort to 97.62% in the 2014 birth cohort (Table S1). The average coverage rate was 96.23% in each birth cohort (2013–2018). While a second dose of the VarV was administered to children aged 4 years, vaccination data for the birth cohort (2016–2018) were lacking. The coverage rate of the second dose of the varicella vaccine ranged from 81.76% in the 2013 birth cohort to 90.47% in the 2014 birth cohort.

### 3.3. Association of Two-Dose VarV Program and PHSMs with the Incidence of Varicella

**Figure 1:**
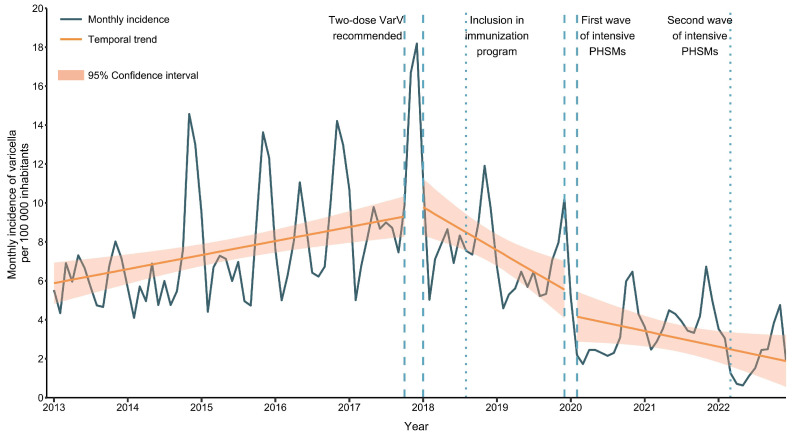
Association of the implementation of the two-dose VarV strategy and PHSMs with the monthly incidence of varicella per 100,000 inhabitants in Minhang District, 2013–2022. The blue line represents the monthly varicella incidence in Minhang, Shanghai, during 2013–2022. The orange slope lines are incidence trends estimated via segmented regression. The orange shading areas are the 95% CIs estimated via segmented regression. The dashed vertical lines represent the transition periods, including the two-dose VarV strategy implementation (November–December 2017) and the first wave of PHSM implementation (January 2020). The dotted vertical lines represent the inclusion of the two-dose VarV strategy in the local immunization program (August 2018) and the second wave of PHSM implementation (March 2022). PHSMs: public health and social measures; VarV: varicella vaccine.

Before the implementation of the two-dose VarV strategy, the monthly incidence of varicella estimated via segmented linear regression was 5.88 cases per 100 000 inhabitants in January 2013, with an increase of 1.02% per month (t = 3.74, *p* < 0.001), reaching 9.31 cases per 100 000 inhabitants in October 2017 (Figure 1). A significant decrease in the monthly incidence trend (−1.84% per month, t = −4.19, *p* < 0.001) was recognized following the implementation of the two-dose VarV program in November 2017. After PHSM implementation in January 2020, the monthly incidence of varicella continued to decrease (−1.44% per month, t = 1.74, *p* = 0.08), but not significantly. Sensitivity analyses showed similar results when not excluding the transition periods (Table S2); however, there was a significant reduction in the incidence after PHSM implementation.

**Figure 2:**
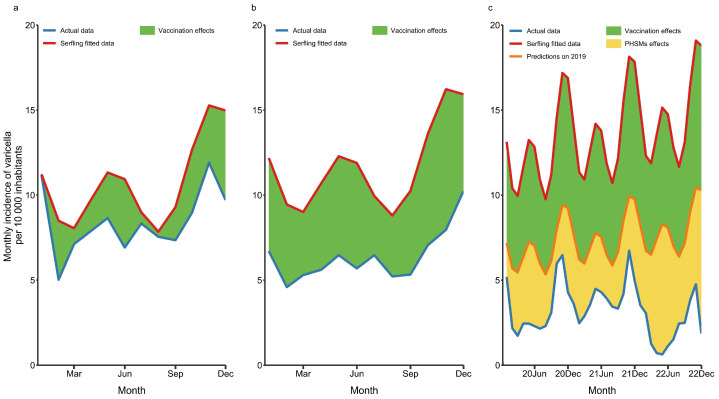
Reduction in the monthly incidence of varicella in Minhang District, 2018–2022. (**a**) Blue line indicates monthly incidence of varicella in 2018. Red line indicates fitted data estimated using Serfling regression. Green shadows indicate the reduction between actual incidence and fitted data, which was a significant reduction of 20.90% (t = −3.14, *p* < 0.001). (**b**) The reduction between actual incidence in 2019 and fitted data was significant, which was 45.25% (t = −13.30, *p* < 0.001). (**c**) Orange line indicates predicted incidence in 2020–2022 on the assumption that identical effect was caused by the two-dose VarV strategy in 2019. A total reduction of 76.51% was observed during 2020–2022, in which 31.26% was attributed to PHSMs (orange shadows). PHSMs: public health and social measures.

Serfling regression was used to predict the varicella incidence from 2018 through 2022 based on the monthly incidence of varicella in Minhang during 2013–2017 (Figure S1). After the implementation of the two-dose VarV strategy, the actual incidence was significantly reduced by 33.08% (t = −8.82, *p* < 0.001) in 2018–2019, 20.90% (t = −4.89, *p* < 0.001) in 2018, and 45.25% (t = −13.30, *p* < 0.001) in 2019, respectively (Figure 2). To estimate the impact of PHSMs during the COVID-19 pandemic, the decrease in varicella incidence caused by the two-dose VarV strategy was assumed to be identical to that in 2019, which was 45.25%. Then, the additional decrease caused by PHSMs was quantified to be 31.26% (t = −27.36, *p* < 0.001), which accounted for 40.86% of the total reduction in varicella incidence (Figure 2). Regarding age-specific influences, the varicella incidence in children aged 0–4 years and 5–19 years was more affected by the implementation of the two-dose VarV strategy compared to that in adults (59.12% and 54.09% vs. 19.49%). However, PHSMs influenced the varicella incidence more in adults than in children (41.71% vs. 16.28% and 30.85%) (Table S3).

**Figure 3:**
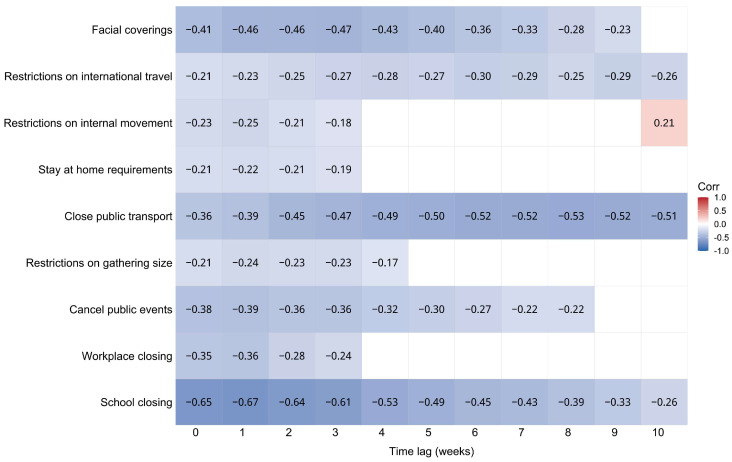
Results of cross-correlation Spearman rank correlation between PHSMs and the number of weekly varicella cases in Minhang District, 2020–2022. Each figure denotes the Spearman rank correlation coefficient between the PHSM in the row and the number of varicella cases at time-shifted intervals of 0–10 weeks. Only significant results are presented (*p* < 0.05). PHSMs: public health and social measures.

The relationship between each PHSM and the number of weekly varicella cases was examined using cross-correlation Spearman rank correlation. As expected, negative correlations were observed for each indicator. Cross-correlation showed similar patterns across indicators, which became increasingly negative and then weakened with more time lags. Among the nine indicators, school closing showed the strongest correlation, with r = −0.67 at a 1-week lag (*p* < 0.001), and three indicators, including closing public transport (r = −0.53 at an 8-week lag, *p* < 0.001), facial coverings (r = −0.47 at a 3-week lag, *p* < 0.001), and the cancellation of public events (r = −0.39 at a 1-week lag, *p* < 0.001) (Figure 3), showed moderately negative correlations. Considering the potential confounders, partial correlations and multiple regression were conducted. The results showed that school closing continued to have the strongest correlation in partial correlation analysis (r = −0.65, *p* < 0.001) (Figure S2) and was the most influential measure (b = −8.03 cases, *p* < 0.001) in multiple regression (Table S4).

## 4. Discussion

The present study explored the impact of a two-dose VarV immunization program and PHSMs on varicella dynamics in Minhang, Shanghai. After the inclusion of the two-dose VarV strategy in the local immunization program, the incidence trend of varicella significantly decreased, with a slope of −1.84% per month (*p* < 0.001) during 2018–2019, which is a substantial change compared to the upward trend in the previous period (slope: 1.02% per month, *p* < 0.001). The incidence decreased by 20.90% in 2018 and 45.25% in the following year. In the PHSM period, the incidence trend of varicella continued to decrease but was nonsignificant, with a slope of −1.44% per month (*p* = 0.08). By assuming the influence of the two-dose VarV immunization program to be identical to the 45.25% reduction observed in 2019, the impact of PHSMs was estimated to be a 31.26% reduction in varicella incidence. More specifically, school closing was found to be most strongly associated with the incidence of varicella (b = −8.03 cases, r = −0.67 with a 1-week lag, *p* < 0.001), and three PHSMs, including closing public transport, facial coverings, and the cancellation of public events, showed moderate correlations.

The results showed that the average coverage rate of the one-dose or first-dose VarV was 96.23% in 2013–2018 birth cohorts and that of a second-dose VarV was 86.06% in 2013–2015 birth cohorts after the introduction of the two-dose VarV program, which is higher than that in other areas of China [28,29,30,31]. The varicella incidence decreased by 45.25% after one year of the two-dose VarV immunization program. A voluntary two-dose VarV strategy has been implemented in Beijing since 2013, which showed that the coverage of a second-dose VarV ranged from 40.1% to 72.9% [31]. The higher coverage rate of VarV in Minhang may be a consequence of the inclusion of VarV in Shanghai’s local immunization program. Since VarV has been offered to children free of charge, vaccination is encouraged for children because it can protect children from disease or at least reduce severe symptoms. Further, the higher coverage may contribute to a greater reduction in varicella incidence compared to that of 37.8% in Beijing [31].

Although declines in varicella incidence occurred across all age groups, children benefited more from the two-dose VarV strategy than adults. The varicella incidence rate was reduced by 59.12% and 54.09% among children aged 0–4 years and 5–19 years, respectively, versus 19.49% in adults. As children aged 4 years and younger were the main targets of the second-dose VarV strategy, this result offered insights that the decline in varicella incidence may be attributed to the two-dose vaccination program. More importantly, VarV vaccination conferred a substantial reduction in varicella incidence in the non-vaccine target groups (children aged 5–18 years and adults aged 19 years and older), which suggests that substantial herd protection could be provided by vaccinating children. This herd protection has been observed for several kinds of vaccines and is crucial for maintaining and improving community and public health.

Moreover, this study found that an additional decrease of 31.26% in varicella incidence may be attributed to PHSMs during 2020–2022. It is lower than that in a previous study that reported a 54% additional decrease caused by social distancing during the COVID-19 pandemic [32]. This may be because this study included the entire pandemic period from 2020 to 2022, whereas the previous study only considered 2020, which was a more strictly managed period. To plan preventive measures for varicella in the post-pandemic era, this study investigated the correlation between each PHSM and the number of varicella cases using the detailed scores of PHSMs recorded during the pandemic. The results showed that school closing with a 1-week lag showed the strongest correlation, while closing public transport and facial coverings showed a moderate correlation, even after excluding confounders. Therefore, a combination of these measures may be a practical supplement to varicella vaccination programs for preventing and controlling varicella outbreaks.

As varicella has not been officially regulated as a notifiable infectious disease in many countries, including China, the actual burden is usually under-reported [9]. In a systematic review for the estimation of the varicella burden in Europe, an important under-reporting in the surveillance system was found, with only <1% (Greece: 6 vs. 109,214 cases) to 51% (Slovenia: 11,074 vs. 21,729 cases) of all cases of varicella reported [33]. In China, it was estimated that the underestimated rate of varicella cases ranged from 3% to 89% at the provincial level, except for Shanghai, the only place in which it was not underestimated [9]. In Shanghai, varicella has been managed as a monitored disease since 2006 and as a notifiable infectious disease since 2018. After more than eight years of regulation, it is unlikely that the routine surveillance of varicella would underestimate the actual incidence in Shanghai. Therefore, this study can provide more complete and reliable results about the impact of a two-dose VarV immunization program on varicella dynamics using real-world data.

There were several limitations in this study. First, although one dose or the first dose of the VarV was found to have a high coverage level in the 2013–2018 birth cohorts, detailed data on the coverage of the second dose of the VarV in the 2016–2018 cohorts were lacking. Second, the relative impacts of the two-dose VarV program and PHSMs were difficult to distinguish, as they coincided during 2020–2022. Additionally, multiple PHSMs were applied simultaneously in this period, making it difficult to understand the relative contribution of each of these measures. However, this study utilized statistical methods such as Serfling regression and partial correlation to quantify their impacts more reliably. Third, as the surveillance data did not provide vaccination information for reported varicella cases, and no identifiable human data were included in this study, we did not match the records between varicella cases and VarV and failed to conduct the test-negative design analysis to evaluate vaccine effectiveness and reveal the epidemic of breakthrough cases. Furthermore, due to the lack of individual-level data, only population-level analyses were performed. Nonetheless, our study used a strong quasi-experimental design to assess the effect of the two-dose VarV strategy, which may minimize the bias. Finally, cost-effectiveness analyses of the two-dose VarV immunization program were not conducted in this study. As it is an important aspect of the inclusion of vaccines in immunization programs, this analysis will be needed in future studies.

## 5. Conclusions

In conclusion, this population-based ecological study, with its long time span, complete surveillance records, and age-specific incidence rates, evaluated the population-level effect of the two-dose VarV immunization program on varicella incidence using real-world data in Shanghai, China. The results suggested that the introduction of the two-dose VarV strategy into the immunization program was associated with a substantial reduction in varicella incidence, and the reduction was larger in children than in adults. Moreover, this study identified that interventions such as school closings, as well as closing public transport and facial coverings, could effectively serve as supplementary measures to help prevent varicella.

These findings provide evidence for countries and regions currently considering the inclusion of varicella vaccines in their routine immunization programs, especially in places where varicella remains a public health problem. Ongoing surveillance of varicella will be needed to describe more fully the impact of varicella vaccination programs and monitor the dynamics of varicella disease.

## Figures and Tables

**Table 1:** Demographic characteristics of reported varicella cases in Minhang District, 2013–2022.

Characteristics	Patients N (%)			
	All Study Periods including Transitions (January 2013–December 2022) ^1^	One-Dose VarV Period (January 2013–October 2017)	Early Two-Dose VarV Period (January 2018–December 2019)	Pandemic Period (February 2020–December 2022)
Total number of cases	19,593 (100)	11,105 (56.68)	4513 (23.03)	2953 (15.07)
Sex				
Female	8937 (45.61)	5039 (45.38)	2096 (46.44)	1353 (45.82)
Male	10,656 (54.39)	6076 (54.71)	2417 (53.56)	1600 (54.18)
Age, years				
Median (IQR)	11 (5–24)	9 (5–22)	12 (7–26)	18 (8–29)
Age Group				
0–4 years	3594 (18.34)	2241 (20.18)	682 (15.11)	529 (17.91)
5–19 years	9505 (48.51)	5695 (51.28)	2177 (48.24)	1037 (35.12)
20–29 years	3782 (19.30)	2041 (18.38)	898 (19.90)	679 (22.99)
30–39 years	2379 (12.14)	1025 (9.23)	675 (14.96)	573 (19.40)
≥40 years	333 (1.70)	103 (0.93)	81 (1.79)	135 (4.57)
Occupation				
Scattered children	2245 (11.46)	1389 (12.51)	437 (9.68)	354 (11.99)
Nursery children	3408 (17.39)	2290 (20.62)	611 (13.54)	285 (9.65)
Students	7748 (39.54)	4393 (39.56)	1888 (41.83)	996 (33.73)
Others	5203 (26.56)	2538 (22.85)	1318 (29.20)	1120 (37.93)
Unknown	989 (5.05)	495 (4.46)	259 (5.74)	198 (6.71)

^1^ Two transition periods were defined, including November to December 2017, when the two-dose VarV strategy was implemented, and January 2020, when PHSMs for COVID-19 were implemented. Abbreviations: PHSMs, public health and social measures; VarV, varicella vaccine.

## Data Availability

The data presented in this study are available on request from the corresponding author.

## Supplementary Materials

The following supporting information can be downloaded at: https://www.mdpi.com/article/10.3390/vaccines11111674/s1. Table S1. Varicella vaccine coverage rate in children born between 2013 and 2018 in Minhang District. Table S2. Effects of the two-dose VarV strategy and PHSMs on varicella incidence trends in Minhang District, 2013–2022. Figure S1. Monthly incidence of varicella cases and predictions using Serfling regression in Minhang District, 2013–2022. Table S3. Estimated reduction in varicella incidence after the two-dose VarV strategy in different age groups in Minhang District, 2018–2022. Figure S2. Partial correlation between PHSMs and the number of weekly varicella cases in Minhang District, 2020–2022. Table S4 Multiple regression of the number of weekly varicella cases on PHSMs in Minhang District, 2020–2022.
