# Supplementary material for: Impact of Varicella Immunization and Public Health and Social Measures on Varicella Incidence: Insights from Surveillance Data in Shanghai, 2013–2022
Source: Vaccines (Basel). 2023 Nov 1;11(11):1674. doi: 10.3390/vaccines11111674 (PMC10674188; doi:10.3390/vaccines11111674)
Supplement: Supplementary file 1 [file vaccines-11-01674-s001.zip › vaccines-2658961-supplementary.pdf]

Supplementary material

Contents

1. **Supplementary Table S1. Varicella vaccine coverage rate in children born between 2013 and 2018 in Minhang District. 2**
2. **Supplementary Table S2. Effects of the two-dose VarV strategy and PHSMs on varicella incidence trends in Minhang District, 2013–2022. .... 3**
3. **Supplementary Figure S1. Monthly incidence of varicella cases and predictions using Serfling regression in Minhang District, 2013–2022. .... 4**
4. **Supplementary Table S3. Estimated reduction in varicella incidence after the two-dose VarV strategy in different age groups in Minhang District, 2018–2022. .... 5**
5. **Supplementary Figure S2. Partial correlation between PHSMs and the number of weekly varicella cases in Minhang District, 2020–2022. .... 6**
6. **Supplementary Table S4. Multiple regression of the number of weekly varicella cases on PHSMs in Minhang District, 2020–2022. .... 7**

Supplementary Table S1. Varicella vaccine coverage rate in children born between 2013 and 2018 in Minhang District.

Birth cohorts	Vaccine coverage rates	
	One dose or first dose	Second dose
2013	96.55%	81.76%
2014	97.62%	90.47%
2015	97.54%	85.95%
2016	97.31%	NA
2017	95.70%	NA
2018	92.67%	NA

Note: The varicella vaccine coverage rates were the proportion of vaccinated children in a birth cohort to all children in that birth cohort. As the second dose of the varicella vaccine was administered to children aged 4 years and older, some birth cohorts (2016–2018) were not qualified for vaccination when data were collected, and thus did not calculate the coverage rates. NA: Not available.

Supplementary Table S2. Effects of the two-dose VarV strategy and PHSMs on varicella incidence trends in Minhang District, 2013–2022.

Incidence trends	One-dose VarV period (January 2013–October 2017)	Early two-dose VarV period (January 2018–December 2019)	Pandemic period (January 2020–December 2022)
Varicella incidence trend	**1.02%*** **	**1.84%*** **	−1.44%
Age-specific incidence trends			
0–4 years	**0.88%*** **	**−2.64%*** **	**−0.72%** **
5–19 years	**1.18%*** **	**−2.40%*** **	**−1.87%* **
≥20 years	**1.04%** **	0.32%	−1.40%
Sensitivity analysis	**1.22%*** **	**−1.95%*** **	**−1.64%* **

Note: Boldface indicates statistical significance (*p<0.05, **p<0.01, *** p<0.001). The trends of varicella incidence were estimated by segmented regression excluding two transition periods, which were November–December 2017 and January 2020. Sensitivity analysis involved segmented regression without excluding transition periods. PHSMs: public health and social measures, VarV: varicella vaccine.

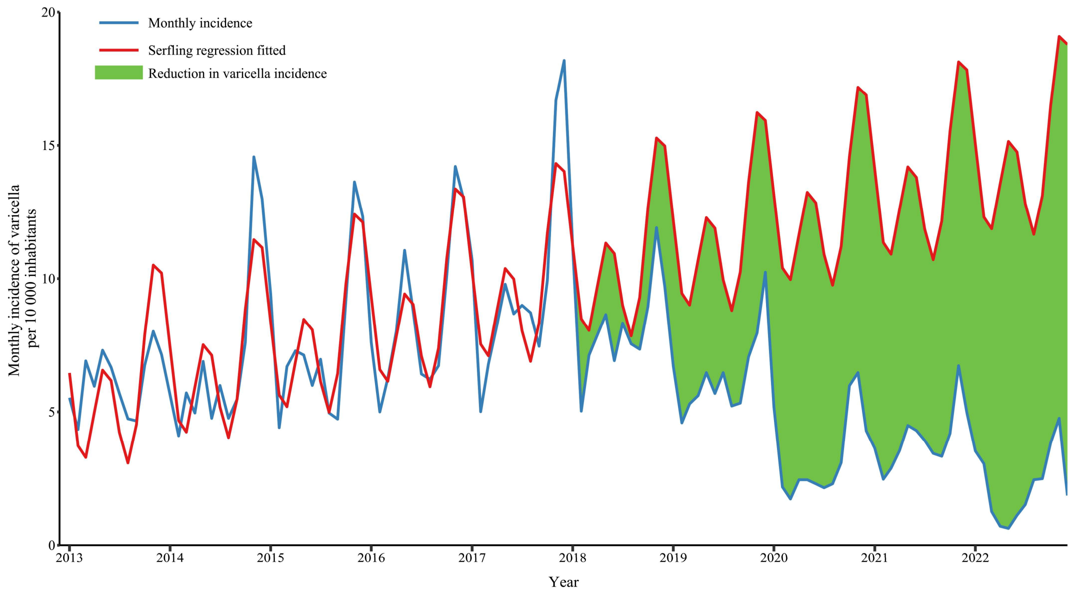

Supplementary Figure S1. Monthly incidence of varicella cases and predictions using Serfling regression in Minhang District, 2013–2022. The blue line represented the actual monthly incidence. The red line represented the fitted data using Serfling regression (equations fitted on the data 2013–2017 and predictions made on 2018–2020). The reduction in the monthly incidence of varicella is demonstrated with green shadows, in which 20.90% in 2018, 45.25% in 2019, and 76.51% during 2020–2022.

Supplementary Table S3. Estimated reduction in varicella incidence after the two-dose VarV strategy in different age groups in Minhang District, 2018–2022.

Age groups	2018	2019	2020-2022[a]
0-4 years	27.66%	59.12%	75.40% (16.28%)
5-19 years	23.56%	54.09%	84.94% (30.85%)
≥20 years	11.06%	19.49%	61.20% (41.71%)
Total	20.90%	45.25%	76.51% (31.26%)

Note: The proportion of reduction in varicella incidence was the difference between fitted data by Serfling regression and actual incidence data. The impacts of PHSMs on varicella incidence were estimated during 2020–2022, on the assumption that the effects caused by the two-dose VarV program was identical to that in 2019. PHSMs: public health and social measures, VarV: varicella vaccine.

[a]Proportion in the brackets represents the estimated effect due to PHSMs implemented during 2020–2022.

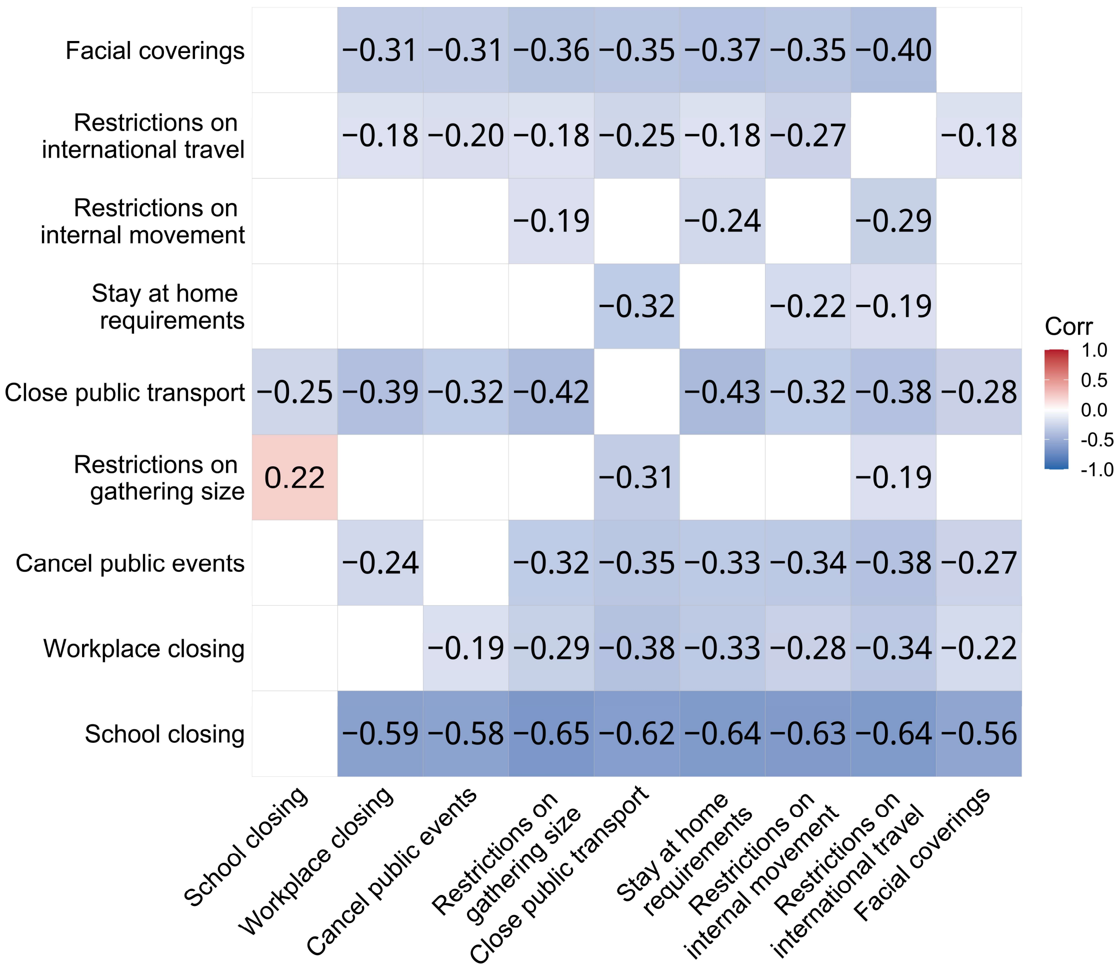

Supplementary Figure S2. Partial correlation between PHSMs and the number of weekly varicella cases in Minhang District, 2020–2022. Each figure denotes the Spearman rank correlation coefficient between the PHSM in the column and the number of weekly varicella cases while controlling for variables in the row. Only significant results were presented ($p < 0.05$). PHSMs: public health and social measures.

Supplementary Table S4. Multiple regression of the number of weekly varicella cases on PHSMs in Minhang District, 2020–2022.

Variables	b/cases	t	*p*
School closing	−8.03	−5.59	**<0.001**
Workplace closing	−1.06	−0.57	0.57
Cancellation of public events	1.51	0.78	0.44
Restrictions on gathering size	1.65	1.99	0.05
Close public transport	−2.05	−2.13	**0.04**
Stay at home requirements	−2.60	−1.83	0.07
Restrictions on internal movement	−0.06	−0.03	0.98
Restrictions on international travel	−2.44	−2.23	**0.03**
Facial coverings	0.35	0.21	0.84

Note: Boldface indicates statistical significance ($p < 0.05$). The dependent variable was the weekly number of varicella cases in Minhang District during 2020–2022. The independent variables were the weekly average scores of PHSMs during 2020–2022 obtained from OxCGRT database. PHSMs: public health and social measures.
